# A cellulose/β-cyclodextrin nanofiber patch as a wearable epidermal glucose sensor

**DOI:** 10.1039/c9ra03887f

**Published:** 2019-07-23

**Authors:** Kyu Oh Kim, Geon Jin Kim, Ji Hye Kim

**Affiliations:** Department of Fiber-System Engineering, Dankook University. 152 Jookjeon-ro, Suji-gu Yongin-si Gyeonggi-do 16890 Republic of Korea kokim@dankook.ac.kr +82-10-3023-0095; Department of Fusion System Engineering, Dankook University. 152 Jookjeon-ro, Suji-gu Yongin-si Gyeonggi-do 448-701 Republic of Korea jhkim@dankook.ac.kr +82-31-8005-3597

## Abstract

In this study, we aimed to develop a cellulose/β-cyclodextrin (β-CD) electrospun immobilized GOx enzyme patch with reverse iontophoresis for noninvasive monitoring of interstitial fluid (ISF) glucose levels (0.1–0.6 mM dm^−3^). *In vitro* analysis, performed using a sensor attached to flexible substrates, revealed that the high diffusion coefficient (9.0 × 10^−5^ cm^2^ s^−1^), the linear correlation coefficient (*R*^2^ = 0.998), the detection limit (9.35 × 10^−5^ M), and the linear range sensitivity (0–1 mM) of the sensor (5.08 μA mM^−1^) remained unaffected by the presence of interfering substances (*e.g.*, fructose, sucrose, uric acid, and acetaminophen) at physiological levels. The present results indicate that the new epidermal sensing strategy using nanofibers for continuous glucose monitoring has potential to be applied in diagnosis of diabetes.

## Abstract 2

In this study, we aimed to develop a cellulose/β-cyclodextrin (β-CD) electrospun immobilized GOx enzyme patch with reverse iontophoresis for noninvasive monitoring of interstitial fluid (ISF) glucose levels (0.1–0.6 mM dm^−3^).

## Graphical abstract

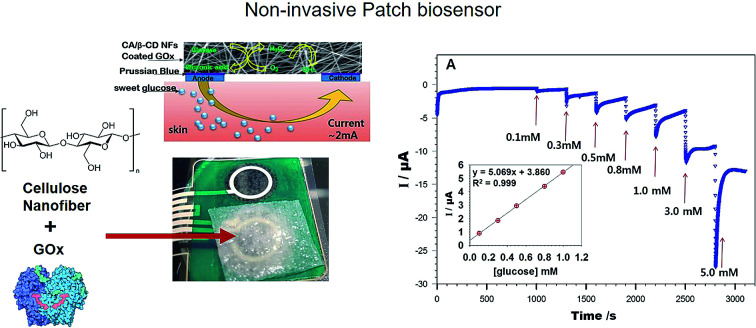

## Introduction

Wearable and mobile technologies have received increasing interest in recent years, which has led to increased efforts toward the development of noninvasive glucose monitoring platforms. Continuous glucose monitoring overcomes the limitations of finger-stick blood sampling, thereby facilitating optimal therapeutic interventions. Reverse iontophoresis was developed by Guy's group for noninvasive glucose monitoring using biosensors.^1–4^ When a small current is applied across the skin, the substance of interest (such as glucose) can be withdrawn from within or beneath the skin to the epidermal surface for detection. However, owing to the barrier properties of skin, the quantity of glucose that can be electroosmotically extracted to the epidermal surface is small, and the corresponding variations in electroosmotically extracted interstitial fluid (ISF) in proportion to those in blood glucose are also small.^5^ Thus, a biosensor platform with a low detection limit, high sensitivity, and high stability for long-term monitoring is warranted to measure glucose levels extracted by reverse iontophoresis.^6^

The use of hydrogels in patch biosensors has been extensively studied.^7^ However, hydrogel-based biosensors have poor sensitivity and a long reaction time and constantly form pores, which renders them difficult to control.^7^ To overcome these limitations, we designed an electrospun nanofiber (NF) with high porosity with micro-scale pore size and high interconnectivity with skin; moreover, the small diameter of the nano-scale fibers provides an increased surface area compared with conventional hydrogels.^8–10^

Cellulose, a biocompatible linear polysaccharide, is chemically stable and possesses high mechanical strength, thus facilitating long-term *in vitro* and *in vivo* stability in sensors.^11^ To develop a functional cellulose-based biosensor, we selected β-cyclodextrin (β-CD), which comprises cyclic oligosaccharides containing seven glucopyranose β-units linked *via* 1,4-glycosidic bonds. Due to their nontoxic and low immunogenicity, CD have extremely attractive biomedical and biomimetic fields. The physical structure of CD is a torus shaped molecule that has a hydrophilic exterior and a relatively hydrophobic core. CD have enables them to form host–guest inclusion complexes including a variety of compounds ranging from small molecules, ions, and proteins to polymers with high selectivity *via* non-covalent interactions.^12–14^ β-CD as an electron shuttle between glucose oxidase (GOx) and an electrode glucose oxidase (GOx) in enzyme-based electrochemical glucose sensors.^15^ Also it known that CD immobilized GOx complexes have long-term stability caused by enzyme denaturation.^16^

In this study, we aimed to fabricate a cellulose/β-CD NF sensor with an electrospun on the reverse iontophoresis electrode Fig. 4(c and d). The electrochemical characteristics of the sensor were elucidated using phosphate buffer solution (PBS, pH 7.4) to determine low glucose concentrations *via* reverse iontophoresis for effective, continuous, noninvasive ISF glucose monitoring.

**Fig. 1:**
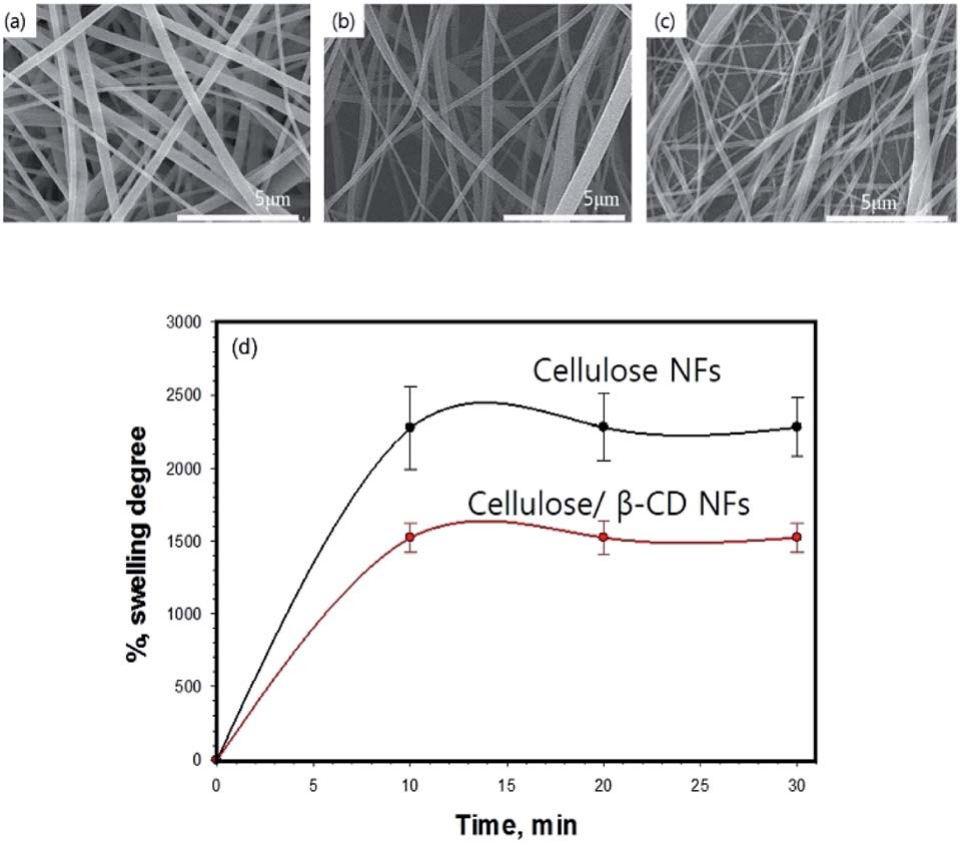
SEM images of cellulose (a), cellulose/β-CD (b), and cellulose/β-CD/GOx (c) nanofibers and the swelling ratio of the cellulose and cellulose/β-CD nanofibers (d).

**Fig. 2:**
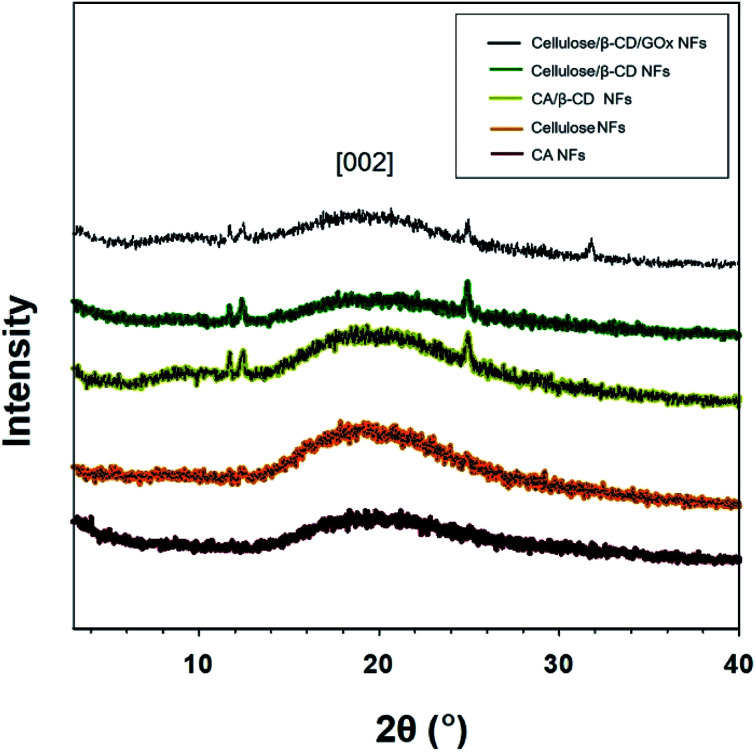
X-ray diffraction patterns of CA, cellulose, CA/β-CD, and cellulose/β-CD/GOx nanofibers.

**Fig. 3:**
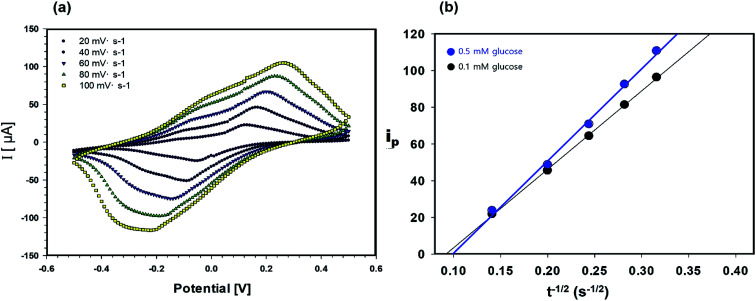
(a) Cyclic voltammetric measurement of current *vs.* scan rate. (b) Plot of *i vs. t*^−1/2^ obtained upon chronoamperometry for cellulose/β-CD/GOx nanofiber biosensors in phosphate buffer solution containing 0.1 and 0.5 mM glucose, with an applied potential of −0.2 V *vs.* an Ag/AgCl electrode.

**Fig. 4:**
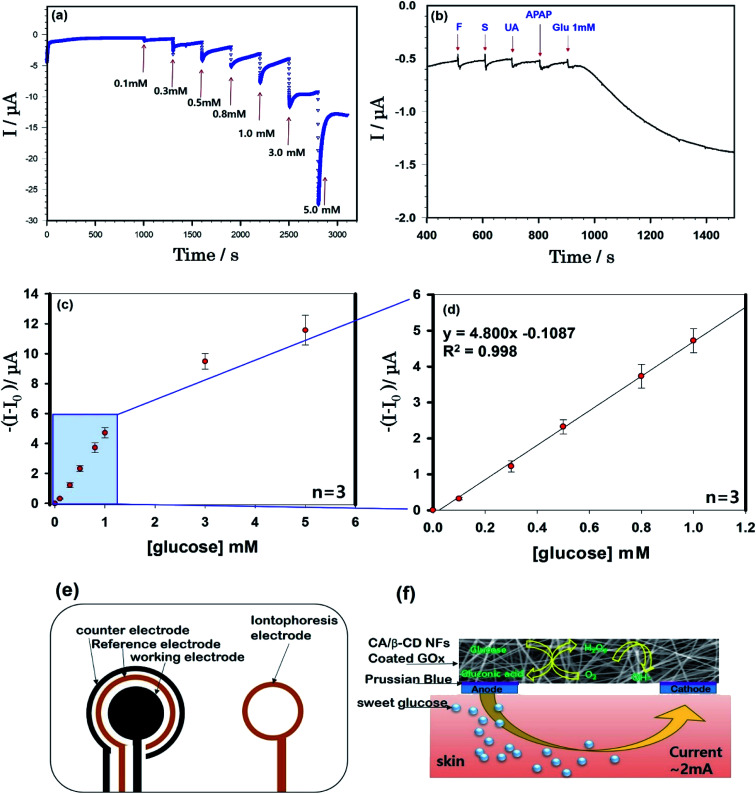
(a) Amperometric responses to successive addition of glucose in 0.1 M phosphate buffer solution at −0.2 V (*vs.* Ag/AgCl). (b) Effect of interfering materials including 1 mM fructose, sucrose, uric acid, acetaminophen, and glucose on the determination of glucose levels. (c) The calibration curve (current *versus* glucose concentration) obtained from the chronoamperometric current response from 0.1 mM to 5.0 mM. (d) The steady-state calibration curve from 0.1 mM to 1.0 mM. (e) A schematic representation of iontophoresis printable electrodes. (f) Mechanism of iontophoresis on the epidermal cellulose/β-CD/GOx NFs glucose sensor.

## Experimental

### Preparation of the cellulose/β-CD NF biosensor

Cellulose acetate (15%, w/v) (CA, Mn = 30 000, Aldrich) and β-CD (2.5 wt%) were dissolved in acetone : *N*,*N*-dimethylformamide (2 : 1, v/v) at room temperature for 24 h. We fabricated the CA/β-CD NF on nonwoven cellulose *via* electrospinning (conditions: TCD, 200 mm; voltage, 15 kV; flow rate, 0.7 mL h^−1^) using a 21 G stainless steel needle. Randomly oriented fibrous scaffolds were accumulated on nonwoven cellulose *via* rotation of a grounding drum (200 rpm). To convert CA to cellulose, deacetylation was carried out with vapors of aqueous 0.05 M NaOH at 40 °C for 20 h. The NFs were then rinsed thoroughly with distilled water until the pH of the NF webs approached 7. Finally, all cellulose NF samples were dried at 25 °C for 24 h. The enzyme solution was mixed with 1.4 g of GOx to in space in 20 mL of 0.1 M, pH 7.4 PBS. The enzyme was then immobilized on the cellulose/β-CD NF using the physical absorption method. To facilitate aging, cellulose/β-CD/GOx NFs were dried for 24 h at room temperature.

### Characterization

The morphology of nanofibrous membranes was characterized by field emission scanning electron microscopy (Nova 400 Nano SEM, FEI) after coating with platinum under a current of 20 mA applied for 120 s. The diameters of the fiber were measured from the SEM micrographs using image analysis software (Image J, National Institutes of Health, USA) and calculated by randomly selecting SEM images of 100–150 fibers. Swelling studies of cellulose and cellulose/β-CD membranes were performed. Briefly, the NFs were weighed in the dry state on an electronic precision balance and then immersed in 10 mL of distilled water. At 0–30 min intervals, the water on the NF surface was gently removed with filter paper, and the volume of water that had been absorbed was determined by weighing the constructs in the swollen condition.

Experiments were performed five times. The swelling ratio of the NFs at equilibrium was calculated using the following eqn (1):

1 Water swelling ratios (*Q*_n_) = (*W*_s_ − *W*_d_)/*W*_d_

where *W*_d_ is the dry weight of the construct and *W*_s_ is the swollen weight of the construct. Advanced thin-film X-ray system-grazing diffractometry was performed, and the patterns of Cu radiation at 12 kW (40 kV, 300 mA) were recorded in the region of 2*θ* from 3 to 50. Electrochemical measurements were obtained with cyclic voltammetric measurements (three-electrode system on an IM6ex electrochemical workstation, Zahner, Germany), using carbon‖Ag/AgCl‖carbon: working‖reference‖counter electrode coated Prussian Blue (Fig. 4(c)) (Engain. Inc) in SATP. Before measurement, cellulose/β-CD/GOx NFs were immersed into 10 mM PBS of pH 7.4 for 10 min for complete swelling of the NFs. The electrons released during glucose oxidation formed a current, which was calibrated to measure the glucose concentration at 0.1–5 mM.

## Results and discussion

We successfully fabricated thin cellulose/β-CD NFs with immobilized GOx. The β-CD complexed with the cellulose chain to form CD inclusion compound channels with polymer guests. For stabilizing enzyme and accurate detection of redox active of cellulose/β-CD nanofiber, a hydrophobic carbon chain of GOx were inserted into β-CD cavity *via* host–guest interactions. For application as biosensor patches, membranes must have high absorbency and surface area and should be able to stably retain moisture during measurement when attached to the patient body. Fig. 1 (upper panel) depicts the morphology of cellulose, cellulose/β-CD, and cellulose/β-CD/GOx NFs. The cellulose/β-CD/GOx NFs had uniform fiber diameter without any bead formation. Pure CA and cellulose have an average diameter of 600 ± 22 nm, whereas CA and cellulose containing β-CD are thinner with an average diameter of 425 ± 120 nm. Fig. 1 (lower panel) shows the swelling ratio of NFs kept in distilled water for 30 min. As cellulose contains many hydrophilic hydroxyl groups (–OH) which allows cellulose microfibrils to form hydrogen bonds with water. The swelling ratio of the cellulose NFs was observed as cellulose/β-CD 26.16 × 10^2^ ± 5.58% and 14.79 × 10^2^ ± 1.12%, respectively. The swelling ratio of the cellulose/β-CD NFs was significantly lower than that of the cellulose NFs. β-CD has higher hydrophobicity and rigidity, it reduced the water absorption capability of the polymer networks.^17^ The cavity of β-CD is limited by hydroxyl groups of different chemical character. Those located at the narrower side come from position 7 of the glucopyranose ring (primary side, R–OH) in cavity, while R–CH_2_OH (secondary side) of β-CD's anhydroglucose units are oriented toward the wider entrance in cavity and therefore less prone to chemical transformation also CD makes them behave as polyols. That is why the hydrophilicity of cellulose/β-CD is lower than that of cellulose NFs.

Fig. 2 shows the X-ray diffraction (XRD) patterns for CA, cellulose, CA/β-CD, cellulose/β-CD, and cellulose/β-CD/GOx NFs. Crystalline cellulose NFs have typical XRD peaks at 2*θ* values of 20.1° and 22.0° corresponding to 4.42 Å and 4.06 Å lattice planes, respectively, of cellulose II.^18,19^ The cellulose NFs exhibit more intensive peaks, indicating enhanced crystallinity compared to CA NFs owing to stronger hydrogen bonding among molecules. NFs with incorporated β-CD displayed three diffraction peaks among many β-CD crystalline peaks. Because the β-CD interrupts hydrogen bonding, the crystallinity of cellulose was weakened.

The permeability of the cellulose/β-CD/GOx NFs, which served as the biosensor electrolyte and as the reservoirs for extracted ISF, is important owing to its shorter warm-up period and enhanced sensitization to glucose. The diffusion coefficient of glucose in the cellulose/β-CD NFs was determined by CV in the potential range of −0.5 to +0.5 V (Fig. 3). The oxidation–reduction peak current (*i*_p_) was determined using the Randles–Sevcik eqn (2):

2 *i*_p_ = (2.69 × 10^5^) × *n*^3/2^ × *D*^1/2^ × *C* × *A* × *υ*^1/2^

where *n* is the number of transferred electrons during the redox reaction, *D* (cm^2^ s^−1^) is the diffusion coefficient, *C* is the molar concentration of glucose (1 and 5 mM), *A* is the electrode surface area (0.785 cm^2^), and *υ* is the scan rate (20–100 V s^−1^). The value of *n* is 1 owing to the following half reaction occurring at the electrode. The profiles are linear (*R* > 0.999), indicating that the sensor is activated along its entire geometric area and that the electrochemical reaction is under diffusion control. The diffusion coefficient for H_2_O_2_ of the cellulose/β-CD/GOx NFs was approximately 9.0 × 10^−5^ cm^2^ s^−1^, which is higher than that obtained using an Os-HRP hydrogel^20^ and a crosslinked PEO hydrogel.^21^ For practical application of the biosensor, optimal permeability of the cellulose/β-CD/GOx NFs facilitates rapid accumulation of extracted glucose and is propitious to the measurement of the extracted glucose.

The epidermal ISF surrounds skin cells through diffusion from the capillary endothelium, which leads to a reliable correlation between blood and ISF glucose levels.^1^ Reverse iontophoresis is carried out by applying a mild current with two skin-worn electrodes to induce ion migration across the skin. Glucose concentrations by extracted ISF are expected to be 1000-fold more dilute relative to blood glucose. Thus, a highly sensitive glucose sensing system is required for accurate blood glucose monitoring.

Fig. 4(a) shows the continuous current responses (current *versus* time) of the cellulose/β-CD/GOx NF biosensor as a function of glucose concentration at room temperature. From the chronoamperometric current response, we have obtained the calibration curve (current *versus* glucose concentration) from 0.1 mM to 5.0 mM (whole concentration) (d) from 0.1 mM to 1.0 mM (low concentration). The cellulose/β-CD/GOx NF biosensor has showed an increasing the standard deviation as the glucose concentration increased. The cellulose/β-CD/GOx NF biosensor less than 1 mM achieved a steady current with a high linear correlation coefficient (*R*^2^) of 0.998 and shown super faster the response time < 3 s as shown in Fig. 4(d). A detection limit of 9.35 × 10^−5^ M was obtained together with a linear range of 0–1 mM, with a sensitivity of 5.08 μA mM^−1^. Although present in ISF at a reasonable level, extracted glucose was estimated to be present at a much lesser level (12.5–125 mM) during each sampling period (15 min);^2^ hence, the detection range and sensitivity of the biosensor are adequate to determine the levels of extracted glucose. Future studies are required to use the sensor fabricated herein to measure 1 mM of interfering samples of fructose, sucrose, uric acid, and acetaminophen. These results indicate that cellulose/β-CD/GOx NF biosensors can selectively detect glucose, as shown in Fig. 4(b).

## Conclusions

In this study, the cellulose/β-CD NF was constructed, which can immobilize GOx on the surface for continuous glucose monitoring patch sensor. This newly developed glucose biosensor demonstrates good absorbency (approximately 14.00 × 10^2^%), the average diameter of 425 ± 120 nm and should be able to stably retain moisture during measurement when attached to the patient body. The developed cellulose/β-CD/GOx NF biosensor with soft and stretchable/flexible materials facilitated uptake of low glucose levels from ISF from the skin *via* high-accuracy reverse iontophoresis for noninvasive, continuous monitoring of glucose. The sensor allowed for highly linear detection of glucose at 0–1 mM with a linear correlation coefficient (*R*^2^) of 0.998, had a short response time < 3 s, a detection limit of 9.35 × 10^−5^ M, sensitivity of 5.08 μA mM^−1^ and exhibited adequate interference rejection. The cellulose/β-CD/GOx NF biosensors fabricated herein suggest potential applications of biosensors for noninvasive, continuous, safe, and sensitive glucose monitoring with low concentrations of extracted glucose.

## Conflicts of interest

There are no conflicts to declare.

## Supplementary Material
